# Motor vehicle fatalities during Memorial Day weekends, 1981–2016

**DOI:** 10.1186/s13104-019-4881-0

**Published:** 2020-01-03

**Authors:** Yuni Tang, Kendra L. Ratnapradipa, Henry Xiang, Motao Zhu

**Affiliations:** 1 Department of Epidemiology, School of Public Health, West Virginia University, Morgantown, WV USA; 2 Department of Epidemiology, University of Nebraska Medical Center, Omaha, NE USA; 3 Department of Pediatrics, College of Medicine, The Ohio State University, Columbus, OH USA; 4 Division of Epidemiology, College of Public Health, The Ohio State University, Columbus, OH USA; 5 Center for Injury Research and Policy, Abigail Wexner Research Institute at Nationwide Children’s Hospital, Columbus, OH USA

**Keywords:** Motor vehicle crash, Fatalities, Memorial Day, Fatality Analysis Reporting System (FARS), Binomial approximation

## Abstract

**Objective:**

Motor vehicle crashes are a leading cause of injury death in the United States, and Memorial Day weekend is one of six holiday periods with an increased number of motor vehicle fatalities in the United States. However, few motor vehicle fatality comparisons were made between Memorial Day weekend and non-holiday periods. Our aims were to determine which day(s) during the holiday had highest motor vehicle fatality risk compared to non-holiday travel and to identify potential risk factors.

**Results:**

Of 43,457 traffic fatalities studied, 15,292 (35%) occurred during the holiday, with Saturday being deadliest but Monday having highest odds of traffic fatality. Both sexes, all years, age < 65, drivers and passengers, rural and urban, and all regions in the United States were at increased risk during the holiday versus non-holiday periods.

## Introduction

### Background

Motor vehicle crashes are a major concern in the United States, with more than 37,000 people killed in 2016, and an increase of nearly 2000 fatalities from 2015 to 2016 [1]. There were 36,560 highway fatalities in 2018, with a decreased 918 fatalities from 2017 to 2018 [2]. Motor vehicle crashes cost an estimated $18 billion in lifetime medical expenses and $33 billion in lifetime work lost in 2012 [3]. Thus, there is an urgent need to improve traffic issues to make roads safer for all road users.

Holidays are times of increased road travel. In 2003, the number of long-distance trips during holiday periods in the United States were higher than the average number for the rest of the year, and motor vehicle is one of the most popular modes of private recreational travel during the holidays in the United States [4]. Memorial Day is a national holiday in the United States occurring the last Monday in May, with the holiday weekend beginning on the preceding Saturday marking the unofficial start of the summer travel season. The Missouri State Highway Patrol found that Memorial Day is one of six holidays with increased traffic crashes [5]. Another report compared injuries and fatalities across six holidays from 1982 to 2001 [6], Memorial Day had the third highest number of fatal crashes. However, neither study compared holiday to non-holiday crashes.

### Objectives

The primary aim of our study was to evaluate the risk of a fatal motor vehicle crash during the Memorial Day weekend and to determine which day(s) had the highest risk. Our second aim was to identify possible risk factors associated with odds of motor vehicle fatality during the Memorial Day weekend.

## Main text

### Methods

#### Data sources

The National Highway Traffic Safety Administration (NHTSA)’s Fatality Analysis Reporting System (FARS) is a national census of fatal traffic crashes in the United States [7]. We used a 1:2 study using time-referenced definition for fatalities during exposed (Memorial weekends) and non-exposed (regular days) period (1981–2016). This study design has been used previously to analyze FARS data [8–12]. The design can adjust for the variation of seasonality and daytime and provide an epidemiologic method for controlling for unmeasured confounders (e.g.: drivers’ education and drive licenses status).

#### Variables

Holiday fatalities were defined as traffic fatalities occurring during the Memorial Day holiday (Saturday through Monday). Non-holiday fatalities occurred during the corresponding 72 h precisely 1 week before and after the holiday. Seven factors were selected for analysis including day of week (Saturday, Sunday, Monday), year groups (5-year group from 1981 to 2010, the final group had 6 years), age (< 18 years old, 18–64 years old, ≥ 65 years old), sex (male, female), role (driver, passenger, pedestrian/other), census region (Midwest, Northeast, South, West), and road location (rural, urban).

#### Statistical methods

Descriptive analysis used Chi-square to compare holiday vs. non-holiday fatality counts and percentages. Because data for non-eventful driving are not available on a date-specific basis, we evaluated odds of holiday fatality using a binomial approximation. This method compares aggregated fatalities for the eventful exposure and eventful non-exposure groups by assuming that the number of non-fatalities are similarly large for Memorial Day weekends and non-holiday periods. An assumed non-eventful driving exposure of one million was used for all study days [10]. In essence, the averaged non-holiday fatalities represent the expected traffic fatalities, and the holidays are the observed fatalities of interest. By dividing the observed by expected fatalities, an odds ratio (OR) and 95% confidence intervals (CI) can be calculated to estimate risk. Because this method relies on aggregated fatalities without considering the non-case exposure status, using a multivariable analytic approach to calculate adjusted odds ratios is not possible. All the analyses were conducted using SAS version 9.4 (SAS Institute, Cary, NC). The statistical significance was defined as *p* < 0.05.

### Results

#### Descriptive data

From 1981 to 2016, 43,557 fatalities occurred during the 3-day Memorial Day weekend (n = 15,292; 35%) and six corresponding comparison days. The average number of deaths per day during the holiday was 142, compared to 132 the week before (n = 14,293) and 128 the week after (n = 13,772). For the holiday, Saturday had the highest number of fatalities (n = 6053; 40%) while Monday (Memorial Day) had the lowest (n = 4104; 26%) (Table 1). By year group, 1986–1990 had the highest holiday fatality count (n = 2466; 16%) and 2006–2010 had the lowest (n = 1998; 13%), but overall counts were stable over time. The majority of fatalities for both holiday and comparison periods were adults and males. During the holiday, 11,745 (77%) of fatalities were adults and 10,879 (71%) were males. Most fatalities during Memorial Day weekend were drivers (n = 8971; 59%). Fatality counts were highest in the South (n = 6692; 44%). More fatalities during each period occurred in urban than rural areas (n = 13,066; 85% vs. n = 2148; 15%).

**Table 1:** Characteristics of individuals involved in fatal crashes during the Memorial Day weekends

	N (%)^1^		*p* value^2^
	Holiday period	Comparison days	
	15,292 (35)	28,065 (65)	
Day of week			< 0.001
Saturday	6053 (40)	11,222 (40)	
Sunday	5135 (34)	9882 (35)	
Monday	4104 (26)	6961 (25)	
Year groups^3^			< 0.001
1981–1985	2299 (15)	4309 (15)	
1986–1990	2466 (16)	4560 (16)	
1991–1995	2079 (14)	3865 (14)	
1996–2000	2090 (14)	3871 (14)	
2001–2005	2238 (15)	4019 (14)	
2006–2010	1998 (13)	3614 (13)	
2011–2016	2122 (14)	3827 (14)	
Age (years)			< 0.001
< 18	1885 (12)	3425 (12)	
18–64	11,745 (77)	21,215 (76)	
≥ 65	1662 (11)	3425 (12)	
Sex			< 0.001
Male	10,879 (71)	20,011 (71)	
Female	4405 (29)	8023 (29)	
Unknown/not reported	8	31	
Role			< 0.001
Driver	8971 (59)	17,087 (61)	
Passenger	4453 (29)	7214 (26)	
Pedestrian/other	1868 (12)	3764 (13)	
Region			< 0.001
Midwest	3327 (21)	6030 (21)	
Northeast	2057 (13)	3544 (13)	
South	6692 (44)	12,523 (45)	
West	3216 (21)	5968 (21)	
Location			< 0.001
Rural	2148 (14)	3768 (13)	
Urban	13,066 (85)	24,136 (86)	
Unknown	78	161	

^1^Missing values and unknowns are not represented in the percentages

^2^The statistical significance was defined as *p *< 0.05

^3^The year group of “2011–2016” has 6 rather than 5 years

#### Main results

**Fig. 1:**
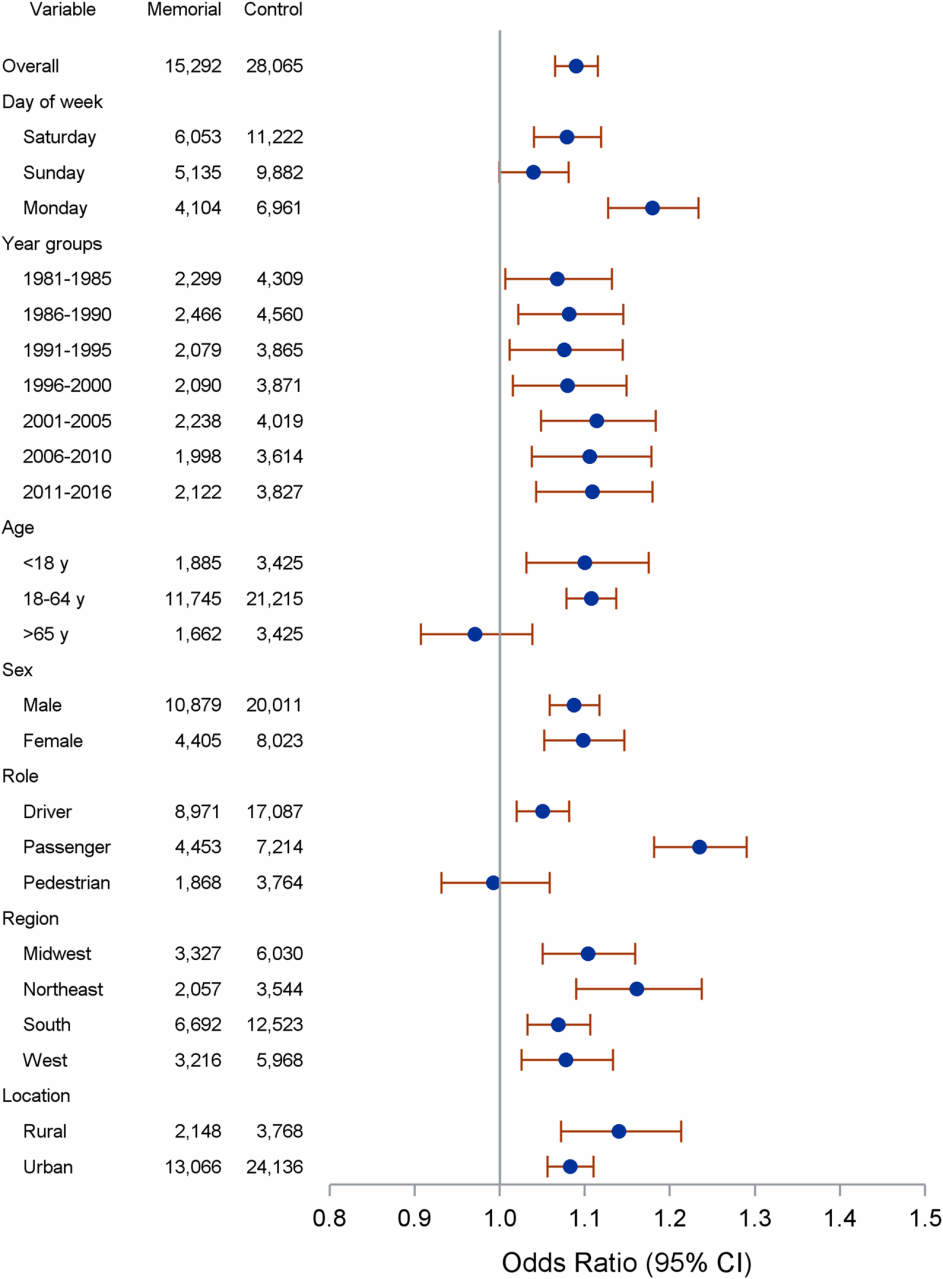
Odds Ratio and 95% CI for Motor Vehicle Fatalities during Memorial Day weekends. This forest plot shows US motor vehicle fatalities from 1981 to 2016. The right side of the forest plot shows a relative increase in risk on Memorial Day weekends versus comparison days computed as odds ratio using the averaged count of comparison fatalities as the referent group. The left side of the forest plot shows all the subgroups of fatalities. Column numbers are fatality counts. Odds ratios greater than 1.00 show an increased risk of crash-related deaths during holiday periods compared to non-holidays, and confidence intervals excluding 1.00 are considered statistically significant at *p *< 0.05

The odds of a motor vehicle fatality occurring during Memorial Day weekend were 9% greater than during the comparison periods (OR: 1.09, 95% CI 1.07–1.12) (Table 2 and Fig. 1). Compared to the non-holiday periods, the odds of fatality on Memorial Day was 18% higher (OR: 1.18, 95% CI 1.12–1.23), and 8% greater on the holiday Saturday (OR: 1.08, 95% CI 1.04–1.12). Analysis of 5-year categories showed they all had increased odds of fatality during Memorial Day weekend versus comparison days.

**Table 2:** Odds ratio and 95% CI for motor vehicle fatalities during Memorial Day weekends

	OR	95% CI	p-value^1^
Total fatalities	1.09	1.07, 1.12	< *0.001*
Day of week			
Saturday	1.08	1.04, 1.12	< *0.001*
Sunday	1.04	1.00, 1.08	*0.05*^2^
Monday	1.18	1.13, 1.23	< *0.001*
Year groups			
1981–1985	1.07	1.01, 1.13	*0.030*
1986–1990	1.08	1.02, 1.15	*0.007*
1991–1995	1.08	1.01, 1.14	*0.021*
1996–2000	1.08	1.02, 1.15	*0.015*
2001–2005	1.11	1.05, 1.18	*0.001*
2006–2010	1.11	1.04, 1.18	*0.002*
2011–2016^3^	1.11	1.04, 1.18	*0.001*
Age (years)			
< 18	1.10	1.03, 1.18	*0.004*
18–64	1.11	1.08, 1.14	< *0.001*
≥ 65	0.97	0.91, 1.04	*0.385*
Sex			
Male	1.09	1.06, 1.12	< *0.001*
Female	1.10	1.05, 1.15	< *0.001*
Role			
Driver	1.05	1.02, 1.08	*0.001*
Passenger	1.23	1.18, 1.29	< *0.001*
Pedestrian/other	0.99	0.93, 1.06	*0.819*
Region			
Midwest	1.10	1.05, 1.16	< *0.001*
Northeast	1.16	1.09, 1.24	< *0.001*
South	1.07	1.03, 1.11	< *0.001*
West	1.08	1.03, 1.13	< *0.001*
Location			
Rural	1.14	1.07, 1.21	< *0.001*
Urban	1.08	1.06, 1.11	< *0.001*

The referent group is fatalities during control days

*OR* odds ratio, *CI* confidence interval

^1^The statistical significance was defined as *p *< 0.05. Statistically significant p-values are italics

^2^We regard p-value = 0.05 as marginally statistical significance

^3^The final year grouping (2011–2016) has 6 rather than 5 years

Odds of traffic fatalities differed by demographics. Adults aged 18-64 experienced 11% higher odds (OR: 1.11, 95% CI 1.08–1.14), and individuals younger than 18 had 10% higher odds of traffic fatalities during the Memorial Day weekend (OR: 1.10, 95% CI 1.03–1.18). Females had 10% increased odds (OR: 1.10, 95% CI 1.05–1.15), and males had 9% increased odds of crash-related fatalities during Memorial Day weekend compared to control days (OR: 1.09; 95% CI 1.06–1.12). Odds of holiday traffic fatalities also differed by role with passengers 17% more likely (OR: 1.23, 95% CI 1.18–1.29) and drivers 5% more likely (or: 1.05; 95% CI 1.02–1.08).

Geographically, people in the Northeast experienced 16% increased odds of fatality during the Memorial Day weekend compared with control days (OR: 1.16, 95% CI 1.09–1.24). Regarding the crash location, the odds of the fatality on rural areas had 14% greater than the control days (OR: 1.14, 95% CI 1.07–1.21), and an 8% increase in urban areas (OR: 1.08, 95% CI 1.06–1.11).

### Discussion

#### Key results

As expected, due to increased traffic volume and longer travel distances during holidays, holiday travel had higher odds of traffic fatality compared to non-holiday periods. Saturday was the deadliest day overall, as well as for holiday and control periods, although Memorial Day Monday had the highest odds of holiday fatality compared to control periods and the odds on Sunday were only marginally significant. Additionally, the increased travel during holidays can explain higher risk of holiday traffic deaths among adults and children (individuals younger than 18). The higher odds of crash-related deaths during holiday periods might reflect children and their families travelling together for longer distances compared to typical work and school commutes. Public campaigns need to make efforts to remind drivers to expect increased congestions and delays during holidays.

Nearly every category we analyzed had increased odds of a holiday traffic fatality, indicating that Memorial Day holiday travel patterns compared to typical travel are not isolated to certain demographic or geographic characteristics. The majority of motor vehicle crash fatalities were in urban areas during both holiday and control days likely due to nearly eighty percent of the U.S. population residing in urban areas [13, 14]. However, rural areas had higher risk of a holiday traffic fatality compared to control days. This may suggest that motor vehicle crashes in rural areas were over-represented during public holidays [15]. Higher risk of traffic fatality in rural areas can reflect increased long-distance travel in these areas during holidays [15, 16].

A holiday, including Memorial Day weekend, is a chance to take off work pressure and enjoy time with friends and family members. Unfortunately, holidays are also associated with increased risky behaviors, such as drinking and other inappropriate driving behaviors [15]. One traffic safety measure is the use of sobriety checkpoints in selected locations to reduce the risk of alcohol-related traffic deaths. However, one study showed that crash fatalities with higher blood alcohol concentration (BAC) threshold remained stable on holidays despite a campaign of targeted enforcement to address the dangers of alcohol-involved driving [17].

The strength of our study was our use of a national census of all crash-related fatalities on public roadways from 1981 to 2016 with comprehensive information for all eligible fatal crashes. We also used an established method of comparing a case period with corresponding double control periods. This method can eliminate multiple unmeasured confounders, clarifies interpretation, and avoids misunderstanding.

In our study, most of the factors with the increased risk during the holiday are the reflection of presumed increased exposure, such as increased holiday travel by distance, time, and increased traffic volume. Therefore, public campaigns are needed to improve road safety of all road users and focus on reminding travelers to expect delays and increased congestion.

### Limitations

The main limitation of our study was data availability. The FARS dataset reports fatal crashes and therefore does not provide estimates of uneventful driving. National travel exposure data, such as estimated vehicle miles or time travelled, are annual estimates and not sensitive enough for date-specific comparisons. A traditional case–control study would have information for the health outcome (case vs. control, in our study fatality vs. non-fatality), as well as exposure status (e.g., holiday vs. non-holiday). Using FARS, we were able to define case exposure status but were missing information for all controls. Therefore, a traditional multivariate regression analysis to calculate adjusted odds ratios was not possible.

Table 2 and Fig. 1 report crude odds ratios calculated using the binomial approximation method, which is a relatively new analysis approach relying on aggregated fatality counts without non-case exposure status [8]. The method has been subsequently validated and elaborated [11, 12]. The method assumes the “controls” of non-eventful driving are similarly large for each exposure status despite increased traffic during holiday periods. The US Department of Transportation estimated that 3.2 trillion highway miles were travelled in 2017 [18]. As a daily average, this equates to 8.8 billion miles per day, so the assumption of similar non-eventful exposure for holiday and non-holiday periods likely holds.

## Data Availability

The FARS dataset was derived from the Internet, at ftp://ftp.nhtsa.dot.gov/FARS and may also be accessed on the web at http://www.fars.nhtsa.dot.gov. The derived data are made available with the article.

## Abbreviations

NHTSA: National Highway Traffic Safety Administration
FARS: Fatality Analysis Reporting System
OR: odds ratio
CI: confidence intervals
BAC: blood alcohol concentration
